# Commentary: Tricuspid valvectomy: A challenge for a cardiac surgeon

**DOI:** 10.1016/j.xjtc.2022.02.005

**Published:** 2022-02-21

**Authors:** Charles C. Canver

**Affiliations:** Cardiovascular and Thoracic Surgery, Lake Regional Health System, Osage Beach, Mo

**Figure:**
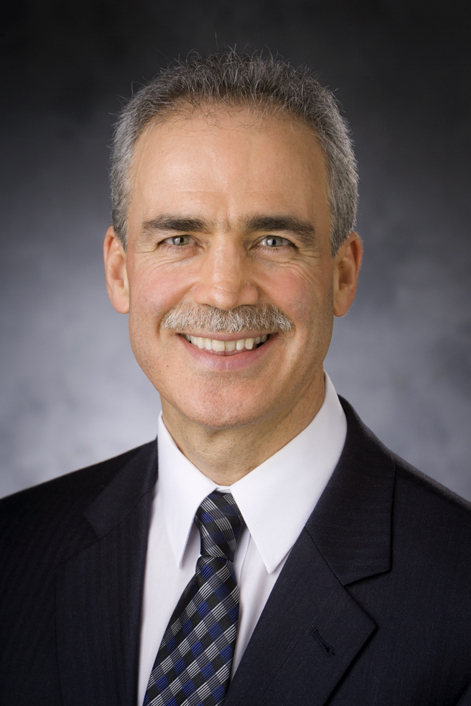
Charles C. Canver, MD, MBA

**Central Message**

Tricuspid valvectomy occasionally might be a temporizing bridge treatment in extremely ill and hemodynamically unstable patients who are prepared for eventual tricuspid valve repair/replacement.

See Article page 65.

Three decades ago, tricuspid valvectomy was the last resort in certain cases of infective endocarditis associated with septic shock in patients with mostly intravenous illicit drug addiction. Excision of infected tricuspid valve is well known to all the cardiac surgeons. The topic is perhaps timely, as recreational drug use has become an alarming national epidemic. In the recent years, the feasibility of tricuspid valve repair or tricuspid valve replacement has been compared with the valve excision option.^1^ In that study, patients with tricuspid valve endocarditis who undergo tricuspid valve excision, repair, and replacement have had comparable outcomes. In consideration of surgeon selection bias, which procedure is superior to other 2 surgical options remain elusive. The matter is furthermore confounded by the high rate of loss to follow-up in these specific subsets of patients. The challenge for a cardiac surgeon is to identify which patient with tricuspid endocarditis is most likely to tolerate tricuspid valve excision instead of repair or replacement. Three decades of symptom-free interval as described in this case report is insufficient to convince any cardiac surgeon for choosing tricuspid valvectomy as the preferred procedure. The patient described in the case report probably had limited endocarditis with a less-virulent micro-organism in the absence of any left-sided heart disease or pulmonary hypertension.^2^ However, the presented patient has probably adopted healthy life choices and stopped using illicit drugs, as he remained free of any recurrent infections. In my opinion, the current recommendation for surgical treatment of tricuspid valve endocarditis is adequate debridement of the infected valve apparatus and treatment of the identified primary source of the infection. If a repair is impossible, a serious consideration should be entertained for tricuspid valve replacement using a bioprosthesis. In a rare occasion, a tricuspid valvectomy might be a temporizing bridge treatment in extremely ill and hemodynamically unstable patients who are prepared for eventual tricuspid valve repair/replacement. Reliable further study of long-term outcomes and survival is warranted in advocacy of any surgical procedure as the choice of surgical treatment for patients with tricuspid endocarditis.
